# Effect of dietary oils from various sources on carbohydrate and fat metabolism in mice

**DOI:** 10.29219/fnr.v64.4287

**Published:** 2020-08-13

**Authors:** Anna Altberg, Ran Hovav, Nava Chapnik, Zecharia Madar

**Affiliations:** 1 Institute of Biochemistry, Food Science and Nutrition, Robert H. Smith Faculty of Agriculture, Food and Environment, The Hebrew University of Jerusalem, Rehovot, Israel; 2 Department of Field and Vegetable Crops, Plant Sciences Institute, ARO (Volcani Center), Bet Dagan, Israel

**Keywords:** oleic acid, soybean oil, gene expression, peanut oil, D7 oil, triglycerides

## Abstract

**Background:**

Dietary oils differ in their fatty acid composition and the presence of additional microcomponents (antioxidants, etc.). These differences are thought to invoke different biochemical pathways, thus affecting fats and carbohydrates metabolism differently. Olive oil (OO) and soybean oil (SO) are common vegetable oils in the local cuisine. Peanuts oils of local varieties are viewed as potential sources of dietary vegetable oils, especially in the food industry.

**Objective:**

We examined the effect of four different dietary vegetable oils on carbohydrate and lipid metabolism in mice. The selected oils were OO, high in oleic acid, extracted from cultivated high oleic acid peanut (C-PO), regular peanut oil (PO), and SO.

**Design:**

In this study, 32 male C57BL/6J mice were randomly divided into four groups (*n* = 8 in each group) and were fed with four different diets enriched with 4% (w/w) dietary vegetable oils (OO, C-PO, PO, or SO). After 10 weeks, the mice were sacrificed. Western blot was used to examine proteins such as phospho-AMP-activated protein kinase (p-AMPK), ace-tyl-CoA carboxylase (ACC), cluster of differentiation 36 (CD36), and Sirtuin 1 (SIRT1), whereas real-time polymerase chain reaction (PCR) was used to examine the expression of sterol regulatory element-binding protein-1c (SREBP-1C), fatty acid synthase (FAS), glucose-6-phosphatase (G6Pase), and CD36 transcripts.

**Results:**

In mice-fed SO, lipid accumulation was predominately in adipose tissue, accompanied a tendency decrease in insulin sensitivity. Mice-fed OO had lower plasma triglycerides (TG) and increased hepatic CD36 gene expression. The C-PO group presented lower messenger RNA (mRNA) levels in the liver for all examined genes: SREBP-1c, FAS, G6Pase, and CD36. There were no significant differences in weight gain, plasma cholesterol and high-density lipoprotein (HDL) cholesterol levels, hepatic ACC, SIRT1, AMPK, and CD36 protein levels or in liver function among the diets.

**Discussion:**

It seems that as long as fat is consumed in moderation, oil types may play a lesser role in the metabolism of healthy individuals.

**Conclusion:**

This finding has the potential to increase flexibility in choosing oil types for consumption.

## Popular scientific summary

- The effect of dietary oils from various sources on carbohydrate and fat metabolism in mice was examined.
- It seems that as long as fat is consumed in moderation, oil types may play a lesser role in the metabolism of healthy individuals.

Fats are essential macronutrients required by the body. Along with providing energy, they have essential roles in cell signaling systems and are critical for absorbing and transporting fat-soluble vitamins. Adipose tissue contributes to many critical survival needs: thermogenesis, lactation, immune responses, and fuel for metabolism (1). However, diets rich in fat can lead to obesity, increased inflammatory markers, insulin resistance, non-alcohol fatty liver disease (NAFLD), and so on (2–5). Fat-rich diets not only lead to NAFLD but can also impair carbohydrates metabolism, manifesting in impaired glucose tolerance curves (6, 7), alterations in gut microbiota (8), and disrupt molecular systems responsible for signaling to the liver (9).

Studies suggest that the quality of fats, more than the quantity, has crucial effects on health (10, 11). Saturated fat intake is associated with increased cholesterol levels, changes in lipid profiles, and increased liver weight. In contrast, monounsaturated fatty acids (MUFAs) and polyunsaturated fatty acids (PUFAs) have been shown to positively impact plasma cholesterol levels in rats (2). Consumption of saturated fats can also lead to glucose intolerance in mice, whereas switching to a diet rich in MUFA improves glucose tolerance (12).

Olive oil (OO), which is rich in MUFA (oleic acid), is considered to be beneficial for health (13). High-MUFA diets have been found to lower both plasma cholesterol and triacylglycerol concentrations (14). Significant associations between higher intakes of OO and reduced risk of all-cause mortality, cardiovascular events, and stroke have been documented in a systematic review and meta-analysis of cohort studies (15). Recently, the FDA recommended consuming oils with high levels of oleic acid to reduce cardiovascular disease (CVD) risk (16).

Risk factors associated with CVD development, such as dyslipidemia, impaired vascular function, and hypertension, can be improved with regular tree nut and peanut consumption (17), and therefore cultivating peanut varieties rich in oleic acid has become popular. The high concentration of oleic acid increases oil stability and decreases oxidation rates (18). In addition, the unique fatty acid profiles of these peanut cultivars are very similar to those of OO. This suggests that they might have similar effects on health. Peanut oils (PO), and especially refined POs, have been found to be safe for use, even among people who are allergic to peanuts (19). All of the above implies that there is potential for POs rich in oleic acid, to positively impact health.

Different types of oils can uniquely impact gene and protein expression in the liver, especially those related to carbohydrate and fat metabolism. Analysis of key genes and proteins can provide a good indication of the active biochemical pathways in the body. For example, sterol regulatory element-binding protein-1c (SREBP-1c) is a key regulatory transcription factor in fatty acid synthesis. It increases gene expression that promotes fatty acid synthesis, such as acetyl-CoA carboxylase (ACC) and fatty acid synthase (FAS) (20). AMP-activated protein kinase (AMPK) is another important enzyme. It plays a role in cellular energy homeostasis (21). Activation of AMPK promotes catabolic pathways to generate more ATP (22). Other key metabolic genes and proteins are Sirtuin 1 (SIRT1), cluster of differentiation 36 (CD36), and glucose-6-phosphatase (G6Pase) (23–26).

We assumed that different types of oils, having different fatty acid profiles, would have varied metabolic effects on the body. The study aims to investigate the effects of different oils with unique fatty acid compositions on liver fatty acid metabolism in mice models. For this research we selected four different oils: OO and soybean oil (SO), which are common vegetable oils in the local cuisine and two peanuts oils of local varieties, which are viewed as potential sources of dietary vegetable oils, especially in the food industry; D7 oil – cultivated high oleic acid peanut oil (C-PO) and Hanoch oil – conventional Israeli PO.

## Materials and methods

### Experimental animals, diets, and sample collection

All experiments were performed within the Hebrew University of Jerusalem’s guidelines of the Authority for Biological and Biomedical Models and were approved by its Institutional Animal Care Ethics Committee. Male C57 BL/6 J mice, 6–7 weeks old, were purchased from Harlan Laboratories (Jerusalem, Israel). Thirty-two mice were randomly assigned to one of four groups that were fed a C-PO diet (extract from cultivation of Israeli peanut variety Einat, enriched with oleic acid, D7), PO diet (extract from cultivation of typical Israeli peanut variety, Hanoch), OO diet, or SO diet. All diets were based on the AIN-93 M diet, with a few modifications (Table 1). Main change was substitution of the SO used in standard AIN-93 M diet (27) with one of the other oils. None of the new chows was set as a control diet. Our goal was aimed to compare the effect of different oils rather than to compare the effect of the oils used in this study to normal oil (control oil).

**Table 1:** Diet composition of the four mice groups

Composition	Diets							
	C-PO		PO		OO		SO	
	(kcal)	(g)	(kcal)	(g)	(kcal)	(g)	(kcal)	(g)
Casein	56	14	56	14	56	14	56	14
l-methionine	0.72	0.18	0.72	0.18	0.72	0.18	0.72	0.18
Cornstarch	198.28	49.57	198.28	49.57	198.28	49.57	198.28	49.57
Maltodextrin 10	50	12.5	50	12.5	50	12.5	50	12.5
Sucrose	40	10	40	10	40	10	40	10
Cellulose	-	5	-	5	-	5	-	5
D7 oil	36	4	-	-	-	-	-	-
Hanoch oil	-	-	36	4	-	-	-	-
Olive oil	-	-	-	-	36	4	-	-
Soybean oil	-	-	-	-	-	-	36	4
Mineral mix AIN-76	-	3.5	-	3.5	-	3.5	-	3.5
Vitamin mix AIL-93-VX	-	1	-	1	-	1	-	1
Choline chloride	-	0.25	-	0.25	-	0.25	-	0.25
BHT	-	0.014	-	0.014	-	0.014	-	0.014
Total	381	100	381	100	381	100	381	100

BHT, butylated hydroxytoluene; C-PO, cultivated high oleic acid peanut oil; PO, peanut oil; SO, soybean oil; OO, olive oil.

Thirty-two mice were randomly assigned to one of four groups that were fed a C-PO diet, PO diet, OO diet, or SO diet. PO and C-PO were extracted from peanuts of these cultivars. All diets were based on the AIN-93 M diet with a few modifications.

Hanoch oil (PO) and D7 oil (C-PO) were extracted from peanuts of these cultivars by a cold press method using a KOMET Twin Screw Vegetable Expeller DD 8G device in the Dr. Ran Hovav Lab. SO and OO were purchased from the local supermarket. The chow for the four diets was prepared in our laboratory and was identical, except for the added oil. The mice were housed in a controlled environment (12/12 h light/dark cycle, 22–24°C) with ad libitum access to food and water. After 9 weeks on the diets, the mice fasted for 12 h and were subjected to an oral glucose tolerance test (OGTT). Before the OGTT, the mice were weighed and marked and were given d-glucose (3 g/kg body weight) by gavage. Glucose levels were monitored at 0, 30, 60, and 120 min after glucose loading. A glucometer-ACCU-CHEX (ROCH) was used to measure glucose levels in blood drawn from the tail tip. After 10 weeks on the experimental diets, the mice fasted for 12 h, their body weights were recorded, and they were sacrificed in random order by an isoflurane overdose. Blood was collected from the vena cava, and plasma was obtained by centrifugation at 5600 × g at 4°C for 10 min, and stored at −20°C. Epididymal adipose tissue was removed, weighed, placed in liquid nitrogen, and stored at −75°C. Liver tissue was collected, weighed, minced in liquid nitrogen, and stored at −75°C.

### Lipid profiles

Oils lipid profiles were determined by gas chromatography method. Total lipids were extracted from oils samples using a protocol adapted from the cold extraction procedure developed by Folch (28). For gas chromotography (GS) analysis, fatty acid methyl esters were generated. Lipid analysis was performed with a gas chromatograph (Agilent Technologies CA, USA) equipped with a fused-silica capillary column. Helium was used as the carrier gas. A known amount of C17:0 was added to the samples prior to extraction to determine the fatty acid concentrations. Peak identification was based on relative retention times of the standard.

### Blood parameters

Analyses of plasma lipid profiles and the liver enzymes were performed by American Laboratories (Herzliya, Israel). Plasma samples were obtained by centrifugation of the blood as described above. In vitro test for the quantitative determination of alanine aminotransferase (ALT), aspartate aminotransferase (AST), alkaline phosphatase, cholesterol, and triglycerides (TG) in plasma were performed using Roche/Hitachi cobas c systems.

### Liver lipid content

The liver lipid content was determined using the Folch (29) method for lipid extraction. A small fraction of liver tissue (~100 mg) was homogenized with a chloroform/methanol solution. Later, the samples were centrifuged at 800 × *g* for 10 min until phase separation was achieved. The upper, transparent phase was collected, and another phase separation was performed. The bottom lipid phase was separated, dried, and weighed.

### Plasma insulin levels

Insulin plasma levels were determined using the Rat/Mouse Insulin ELISA Kit, Cat#EZRMI-13K (Merck Millipore, Darmstadt, Germany), according to the manufacturer’s protocol. Total insulin amounts were calculated using the manufacturer’s standard curve.

### RNA extraction and reverse transcription polymerase chain reaction analysis

Total RNA was isolated from liver tissue by using the Tri-Reagent (Sigma-Aldrich, Rehovot, Israel), according to the manufacturer’s protocol. Complementary DNA was prepared with the qScript cDNA synthesis kit (Quanta BioSciences, Gaithersburg, MD, USA). Real-time polymerase chain reaction (RT-PCR) was performed with the 7300 Real-Time PCR System (Applied Biosystems, Foster City, CA, USA), with specific primers (Table 2). Quantitative changes in gene expression were determined by normalizing against β-actin and by using the delta-delta-Ct (ddCt) algorithm (30).

**Table 2:** List of primers used in RT-PCR

Name	Reverse	Forward
β-actin	5’-GGGGTGTTGAAGGTCTCAAA-3’	5’-CTAAGGCCAACCGTGAAAAG-3’
CD36	5’-AAAGGCATTGGCTGGAAGAA-3’	5’-TCCTCTGACATTTGCAGGTCTATC-3’
FAS	5’-GGTGTTTCTCCATTAAATTCTCAT-3’	5’-CTAGAAACTTTCCCAGAAATTTCC-3’
G6pase	5’-AAGAGATGCAGGAGGACCAA-3’	5’-ACTCCAGCATGTACCGGAAG-3’
SREBP-1c	5’-TAGATGGTGGCTGCTGAGTG-3’	5’-GATCAAAGAGGAGCCAGTGC-3’

FAS, fatty acid synthase.

### Protein extraction and western blotting

Total liver tissue protein was extracted using a lysis buffer containing: 20 mM Tris-HCl (pH 7.4), 145 M NaCl, 10% glycerol, 5 mM EDTA, 1% Triton X-100, 0.5% NP-40, 100 mM phenylmethylsulfonyl fluoride (PMSF), 200 mM NaVO4, 5 mM NaF, and 1% protease inhibitor cocktail. Lysates were centrifuged at 8500 × *g* for 15 min at 4°C, and the protein concentration was determined by the Bradford method with bovine serum albumin used as a standard. The samples were subjected to sodium dodecyl sulfate polyacrylamide gel (12%) electrophoresis (SDS_PAGE), after which proteins were transferred onto nitrocellulose membranes. Blots were then incubated with primary antibodies: antirabbit adenosine monophosphate-activated protein kinase (AMPK), antirabbit phosphorylated adenosine monophosphate-activated protein kinase (pAMPK), antirabbit acetyl CoA carboxylase (ACC), antirabbit phosphorylated acetyl CoA carboxylase (pACC) (Cell Signaling Technology, Beverly, MA, USA), antirabbit monoclonal CD36, antirabbit polyclonal Sirtuin 1 (SIRT1) (Abcam, Cambridge, UK), and antimouse β-actin (BD Biosciences, San Jose, CA, USA) and then, after several washes, with secondary goat antirabbit antibodies (Jackson Immuno Research Laboratories, West Grove, PA, USA). The immune reaction was detected by enhanced chemiluminescence, with bands being quantified by densitometry and expressed as arbitrary units.

### Statistical analyses

Values are presented as means ± SE. Analysis of variance (one-way ANOVA) and the Tukey–Kramer honest significant difference post hoc test were used to compare means. When comparing significant differences between only two groups, a Student’s *t*-test was applied. All data were tested for normal distribution prior to applying any statistical test. The significance level was *P* < 0.05 for all analyses, unless otherwise specified. Connecting letters report was used to display the results of the Tukey–Kramer test (*P* > 0.05). Results that share, or are connected by, the same letter do not differ statistically. Results that are not connected by a common letter do differ statistically. Notations of double letters, like ‘ab’, stand for two levels of significance ‘a’ and ‘b’. Asterisks were used for significant differences in the Student’s *t*-test. There is no special meaning for number of asterisks used. JMP 14 Pro software (SAS Institute, Cary, NC, USA) was used for the statistical analyses.

## Results

### Oil lipid profiles

The fatty acid compositions of different oils used in this study are presented in Table 3. Both the C-PO and OOs were rich in MUFA (mainly oleic acid), 77.9 and 82.8%, respectively. The SO was rich in PUFA (mainly linoleic acid, *ω*6), 55.6%. The linoleic acid/α-linolenic ratio (*ω*6/*ω*3) ratio was lowest in OO (6.8%) and highest in the PO (361%). It should be noted that although both C-PO and OO have similar high oleic acid content, the *ω*6/*ω*3 ratio in C-PO is about 6.5 higher than in OO.

**Table 3:** Fatty acid composition of the four oils used*

Fatty acids	% Area			
	C-PO	PO	OO	SO
Pentadecylic acid (C15:0)	0.000	0.000	0.000	0.017
Palmitic acid (C16:0)	5.682	10.236	10.481	10.670
Palmitoleic acid (C16:1 cis-9)	0.079	0.050	0.750	0.078
Margaric acid (C17:0)	0.085	0.096	0.052	0.091
Stearic acid (C18:0)	2.491	3.456	3.079	4.334
Oleic acid (C18:1 cis-9)	81.111	49.198	76.931	25.210
Vaccenic acid (C18:1 trans-11)	0.501	0.416	2.008	1.413
Linoleic acid (C18:2 cis-9,12)	3.056	29.778	4.312	50.509
Linolelaidic acid (C18:2 trans-9,12)	0.000	0.000	0.000	0.070
α-Linolenic acid (C18:3 cis-9,12,15)	0.069	0.082	0.631	4.974
Arachidic acid (C20:0)	1.211	1.514	0.428	0.533
Paullinic acid (C20:1 cis-11)	1.499	0.897	0.264	0.259
Eicosadienoic acid (C20:2 cis-11,14)	0.000	0.018	0.000	0.043
Mead acid (C20:3 cis-8,11,14)	0.020	0.019	0.018	0.037
Behenic acid (C22:0)	2.239	2.365	0.128	0.563
Erucic acid (C22:1 cis-13)	0.114	0.049	0.000	0.000
Tricosylic acid (C23:0)	0.039	0.037	0.025	0.062
Lignoceric acid (C24:0)	1.353	1.325	0.058	0.200
Squalene	0.043	0.033	0.629	0.000
Linoleic acid/α-Linolenic ratio (*ω*6/*ω*3)	44.553	361.178	6.835	10.155
Total saturated fat	13.099	19.028	14.252	16.469
Total trans fat	0.501	0.416	2.008	1.483
Total MUFA	82.803	50.194	77.945	25.547
Total PUFA	3.145	29.897	4.961	55.563

C-PO, cultivated high oleic acid peanut oil; PO, peanut oil; SO, soybean oil; OO, olive oil; MUFA, mono-unsaturated fatty acids; PUFA, polyunsaturated fatty acids.

* Profiles of the four oils were determined by GC using C17:0 as a standard.

### Bodyweight and food consumption

**Fig. 1:**
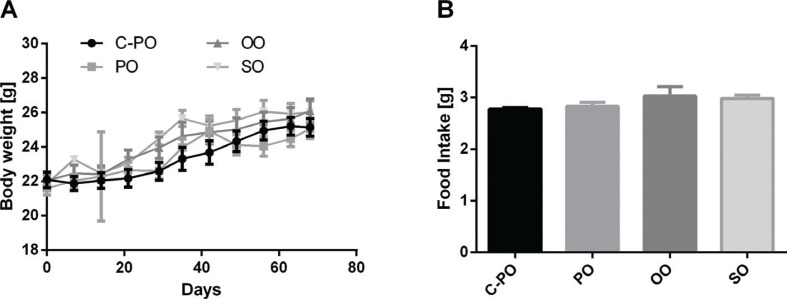
Effects of various oils on bodyweight and food consumption. Male C57 BL/6 J mice aged 6–7 weeks were fed a cultivated high oleic acid peanut oil (C-PO), peanut oil (PO), olive oil (OO), or soybean oil (SO) diet for 10 weeks. (A) Bodyweight was measured weekly for 10 weeks. (B) Average food consumption was measured during a 12-day period. All values are means ± standard error of the means (SEM), *n* = 8.

Weight gain was similar throughout the experiment (Fig. 1A). Similar food intake was observed among all groups of mice regardless of diet composition (Fig. 1B). No statistically significant differences were found among the groups.

### Oral glucose tolerance test

**Fig. 2:**
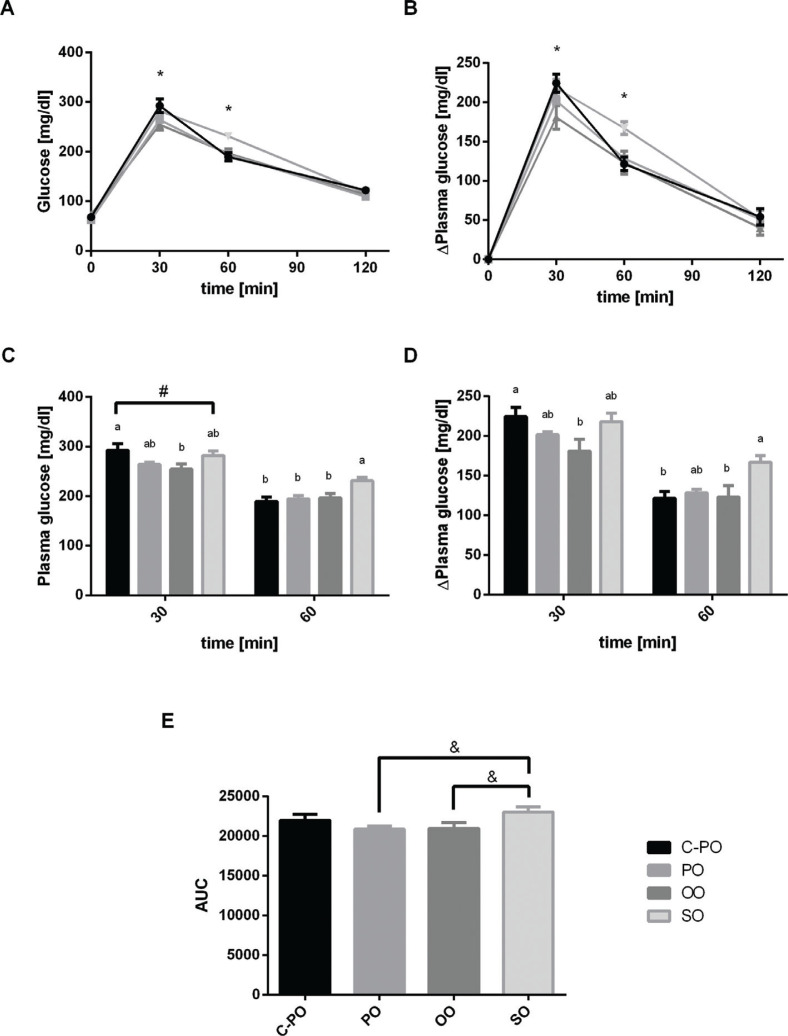
Effect of diet on oral glucose tolerance tests. Male C57 BL/6 J mice aged 6–7 weeks were fed a cultivated high oleic acid peanut oil (C-PO), peanut oil (PO), olive oil (OO), or soybean oil (SO) diet for 10 weeks. Oral glucose tolerance tests (OGTT) were conducted in the 8th week. Mice were administered 0.1 mL of a 30% glucose solution for each 10 g body weight. Blood was sampled from the mouse-tail tips. (A) Glucose tolerance test. Data marked with an asterisk are significantly different (*P* < 0.05) as elaborated, and presented in Fig. C. (B) Difference in plasma glucose levels in comparison to *t* = 0 min. Data marked with * are significantly different (*P* < 0.05) as presented in Fig. D. (C) Plasma glucose levels at *t* = 30 min, *t* = 60 min. Data marked with ‘#’ represent a trend toward significant difference (*P* < 0.06) between the SO diet group and the C-PO diet group in the Student’s *t*-test. (D) Difference in plasma glucose levels at *t* = 30 min, *t* = 60 min. (E) Area under curve (AUC) of the glucose tolerance test. Data marked with ‘&’ represent a trend toward significant differences (*P* < 0.07) between the SO diet group and OO or PO diet groups in the Student’s *t*-test. All values are means ± SEM, *n* = 6–8.

There were no significant differences in glucose levels among the mice fed different diets at baseline and the endpoint of the OGTT. However, intergroup differences were observed at 30 and 60 min. Glucose clearance in the OO group was fastest, while mice-fed SO had the slowest clearance rates (Fig. 2C, D). Also, a tendency (trend) for higher area under the curve (AUC) of OGTT was observed for the SO diet group (23,050 ± 641) when compared to the AUC of the OO diet group (20,991 ± 732) or to the PO group (20,882 ± 387) using the Student’s *t*-test (*P* < 0.07). This implies slower glucose clearance in the SO diet group compare to the PO and OO diet groups.

### Bodyweight, adipose, and liver tissues weight

By the end of the experiment, body weight, fasting glucose levels, and liver tissue weight were similar among the groups. However, adipose tissue weight varied between the diets (*P* < 0.05), 0.48 ± 0.03 for the C-PO group and 0.78 ± 0.09 for the SO group (Table 4.).

**Table 4:** Final body weight, tissue weight and fasting glucose*

	Diets			
	C-PO	PO	OO	SO
Final body weight (g)	23.1 ± 0.4	23.4 ± 0.5	24.4 ± 0.6	23.7 ± 0.4
Fasting glucose level (mg/dL)	162.0 ± 9.6	159.5 ± 10.8	153.6 ± 7.4	138.6 ± 8.8
Adipose tissue (g)	0.48 ± 0.03^b^	0.54 ± 0.04^ab^	0.56 ± 0.08^ab^	0.78 ± 0.09^a^
Liver weight (g)	0.92 ± 0.02	0.90 ± 0.03	0.97 ± 0.04	0.88 ± 0.02

C-PO, cultivated high oleic acid peanut oil; PO, peanut oil; SO, soybean oil; OO, olive oil.

* Male C57 BL/6 J mice aged 6–7 weeks were fed a C-PO, PO, OO, or SO diet for 10 weeks. All values are means ± SEM, *n* = 6–8. Data marked with different letters (a, b) are significantly different (*P* < 0.05).

### Liver and adipose tissue weight to body weight ratio

**Fig. 3:**
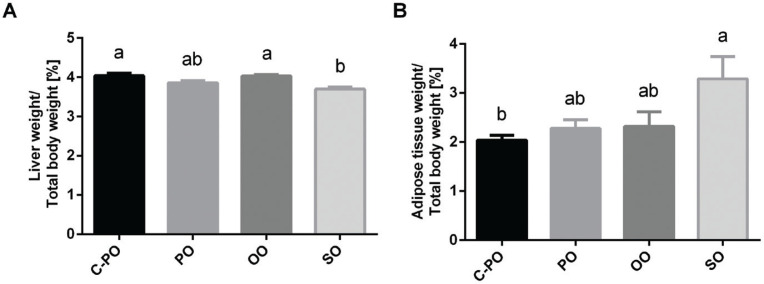
Effect of diets on liver and adipose tissue weight-to-total body weight ratio. Male C57BL/6 J mice aged 6–7 weeks were fed either a cultivated high oleic acid peanut oil (C-PO), peanut oil (PO), olive oil (OO), or soybean oil (SO) diet for 10 weeks. (A) Liver weight to body weight ratio. (B) Adipose tissue to body weight ratio. All values are means ± SEM, *n* = 7–8. Data marked with different letters (a, b) are significantly different (*P* < 0.05).

The SO diet group had significantly lower liver tissue weight-to-body weight ratio (3.70 ± 0.05) (Fig. 3A) and the highest adipose tissue weight-to-body weight ratio (3.29 ± 0.45) (Fig. 3B), *P* < 0.05, while the C-PO diet group had the highest liver tissue weight-to-body weight ratio (4.04 ± 0.07) (Fig. 3A) and the lowest adipose tissue weight-to-body weight ratio (2.04 ± 0.10) (Fig. 3B), *P* < 0.05.

### Lipid accumulation in the liver

**Fig. 4:**
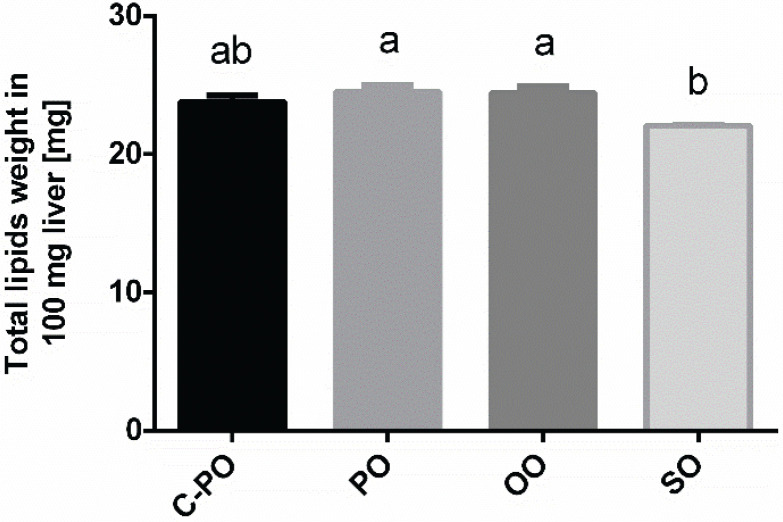
Effect of diets on lipid accumulation in the liver. Male C57 BL/6 J mice aged 6–7 weeks were fed a cultivated high oleic acid peanut oil (C-PO), peanut oil (PO), olive oil (OO), or soybean oil (SO) diet for 10 weeks. The lipid content was determined using the Folch lipid extraction method from 100 mg liver tissue. All values are means ± SEM, *n* = 7–8. Data marked with different letters (a, b) are significantly different (*P* < 0.05).

Lipid accumulation in the liver tissue, also known as ectopic lipid accumulation (Fig. 4) was lowest in the SO diet group (22.07 ± 0.06 mg) compared to other groups (*P* < 0.05).

### Effect of the oils on serum lipids and liver biomarkers

Serum lipids and liver biomarkers are shown in Table 5.There was no significant difference in the AST levels between the diets, and there was no correlation between ALT and alkaline phosphate levels, although significant intergroup differences were found. In addition, plasma lipid profiles showed a similarity in total cholesterol and high-density lipoprotein (HDL) cholesterol levels among all the diet groups. However, TG levels were lowest (64.67 ± 3.47 mg/dL) in the OO group (*P* < 0.05).

**Table 5:** Effect of the diets on serum lipid profiles and liver biomarkers*

	Diets			
	C-PO	PO	OO	SO
Triglycerides (mg/dL)	82.67 ± 6.01^ab^	89.80 ± 4.02^a^	64.67 ± 3.47^b^	90.80 ± 9.01^a^
Cholesterol (mg/dL)	121.60 ± 6.21	117.33 ± 6.43	118.67 ± 5.81	115.40 ± 2.25
HDL cholesterol (mg/dL)	106.22 ± 4.05	110.00 ± 4.77	110.45 ± 5.06	109.40 ± 3.37
Alk Phos (U/L)	100.20 ± 2.96^a^	82.17 ± 2.48^b^	86.00 ± 4.28^b^	77.60 ± 3.44^b^
AST (U/L)	52.80 ± 7.56	53.00 ± 3.00	49.33 ± 6.46	44.60 ± 2.77
ALT (U/L)	16.40 ± 2.62^b^	24.33 ± 2.36^a^	17.20 ± 0.58^ab^	16.00 ± 1.14^b^
Insulin (ng/mL)	1.09 ± 0.06	1.25 ± 0.11	1.43 ± 0.24	1.15 ± 0.04

C-PO, cultivated high oleic acid peanut oil; PO, peanut oil; SO, soybean oil; OO, olive oil; HDL, high-density lipoprotein; AST, aspartate aminotransferase; ALT, alanine aminotransferase,

* Male C57 BL/6 J mice aged 6–7 weeks were fed a C-PO, PO, OO, or SO diet for 10 weeks. All values are means ± SE, *n* = 3–6. Data marked with different letters (a, b) are significantly different (*P* < 0.05).

### Effect of the oils on mRNA gene expression involved in carbohydrate and lipid metabolism

**Fig. 5:**
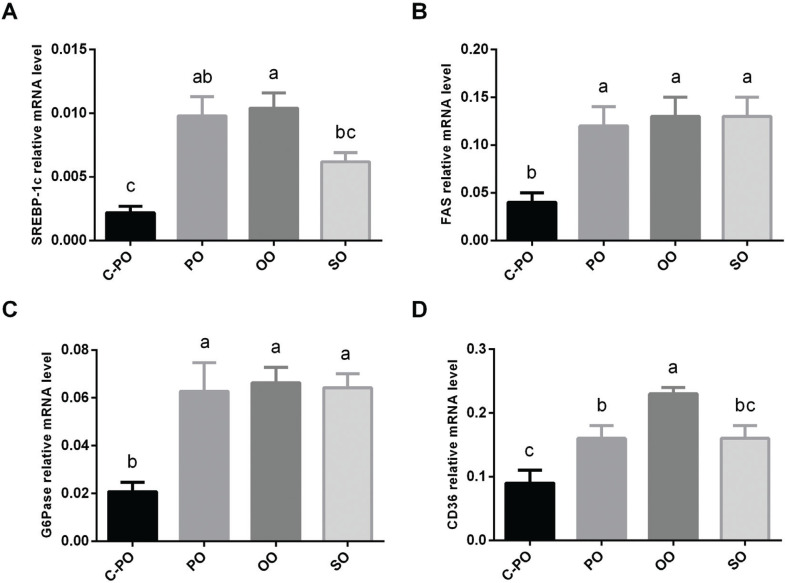
Effects of the diets on gene expression involved in carbohydrate and lipid metabolism. Male C57 BL/6 J mice aged 6–7 weeks were fed a cultivated high oleic acid peanut oil (C-PO), peanut oil (PO), olive oil (OO), or soybean oil (SO) diet for 10 weeks. mRNA levels of the following genes: (A) sterol regulatory element-binding protein-1c (SREBP-1c), (B) fatty acid synthase (FAS), (C) glucose-6-phosphatase (G6Pase), and (D) cluster of differentiation 36 (CD36) were measured using RT-PCR and normalized to β-actin mRNA levels using the ddCt algorithm. All values are means ± SEM, *n* = 7–8. Data marked with different letters (a, b) are significantly different (*P* < 0.05).

mRNA levels of SREBP-1c, FAS, G6Pase, and CD36 genes were significantly lower for the C-PO diet group. In addition, SREBP-1c and CD36 mRNA levels were significantly higher in the OO diet group (Fig. 5).

**Fig. 6:**
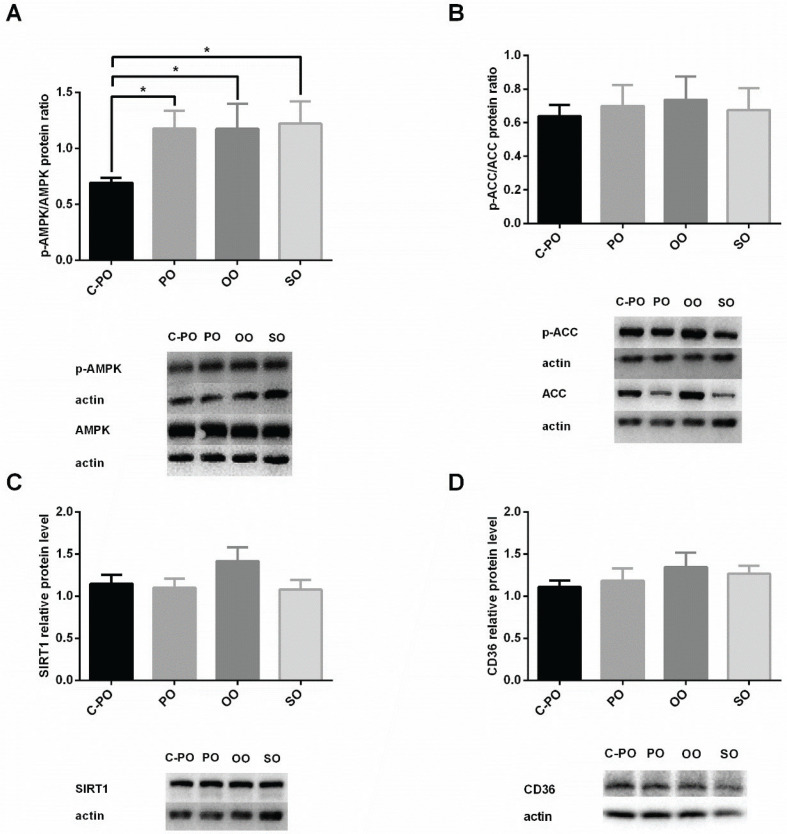
Effect of the oils on protein expression levels relevant to carbohydrate and lipid metabolism. Male C57 BL/6 J mice aged 6–7 weeks were fed a a cultivated high oleic acid peanut oil (C-PO), peanut oil (PO), olive oil (OO), or soybean oil (SO) diet for 10 weeks. Protein expression levels were determined using the western blot method on liver tissue, using actin levels to normalize the values. (A) p-AMPK/AMPK proteins level ratio. *A significant difference (*P* < 0.05) of the C-PO diet compared to other diets in a Students’ *t*-test. (B) p-ACC/ACC protein level ratios, (C) Sirtuin 1 (SIRT1) relative protein levels, (D) cluster of differentiation 36 (CD36) relative protein levels. All values are mean ± SEM.

### Effect of different oils on protein expression levels relevant to carbohydrate and lipid metabolism

The p-ACC/ACC ratio and protein levels of SIRT1 and CD36 showed no significant differences between the groups. The p-AMPK/AMPK ratio showed no significant differences using a Tukey’s Kramer test. However, there was a significant difference (*P* < 0.05) between the C-PO diet and the other diets when analyzed using the Student’ *t*-test.

## Discussion

Metabolic syndrome is a cluster of conditions that occur together, increasing risk of heart disease, stroke, and type 2 diabetes. These conditions include increased blood pressure, high blood sugar, excess body fat around the waist, and abnormal cholesterol or TG levels.

This study aimed to evaluate the effect of different oils with different fatty acid profiles on specific metabolic responses in the metabolism of carbohydrates and fats in mice for better understanding effect of dietary oils on metabolic syndrome. The specific oils studied were the sole source of fat in the diet and the only modified variable among the diets.

Linoleic acid and α-linolenic acid are precursors for essential fatty acids *ω*6 and *ω*3 correspondingly. Lipid analysis showed that the linoleic acid to α-linolenic acid ratio was 44.2% for C-PO (peanuts with high oleic content), 361% for PO, 6.8% for OO, and 10.2% for SO (Table 3). It has been reported that high *ω*6-to-*ω*3 ratio promotes the pathogenesis of many deceases, including CVDs (31). In contrast, increasing *ω*3 levels and decreasing *ω*6-to-*ω*3 ratios are beneficial in the prevention and treatment of coronary artery disease (31). The *ω*6-to-*ω*3 ratio was 6.5 times higher for the C-PO compared to OO. This may be the reason for the differences between the oils and their effect on metabolism. It should be noted that both C-PO and OOs have overall similar lipid profiles and both contain high concentrations of oleic acid.

In this study, all the mice had similar food consumption levels, and weight gain was similar for all groups. However, compared to the other three diets, SO led to higher adipose tissue weight gain, higher adipose tissue weight-to-body weight ratio, and lower liver tissue weight-to-body weight ratio, as well as lower lipid accumulation in the liver tissue. This suggests that the SO metabolic pathways led to fat accumulation in adipose tissue rather than in liver tissue. This effect could be attributed to the unique SO lipid profile which is rich in PUFA (mainly *ω*6) and poor in MUFA compared to other oils. In contrast, in the OO group, liver tissue weight-to-body weight ratio and lipid accumulation in the liver tissue were the highest, suggesting that OO promotes lipid accumulation in the liver, as was also found by other studies (32).

Plasma cholesterol and HDL cholesterol levels were similar for all groups. Interestingly, plasma TG levels were lower in the OO group compared to other groups. This may indicate that in OO-rich diets, TG are more likely transported to various tissues such as liver, adipose, and muscle tissues. The C-PO group had lower TG levels compared to the PO and SO groups. Both olive and C-POs are rich in oleic acid. Previous studies have shown that oleic acid lowers cholesterol levels in plasma (33, 34). There is no consensus regarding oleic acid’s effect on plasma TG levels. Some studies have shown that oleic acid lowers plasma TG levels (15), while in other studies this effect was not observed (34).

Analysis of the liver enzymes indicates that there were no significant pathologies in any of the groups, and the diets had similar effects on the liver. This is considered to be normal in diets with 4% (w/w) fat.

Both fasting glucose levels and blood insulin levels were found to be similar among the groups. However, glucose plasma clearance rates varied. Sixty minutes after glucose intubation, plasma glucose levels were significantly higher in SO-fed mice compared to other diets. Also, a trend was seen for the AUC value (*t*-test, *P* < 0.07), when comparing the SO group to the PO or OO groups. These findings suggest that mice fed a SO diet were less sensitive to insulin, which may lead to slower glucose clearance from the blood compared to other diets. This is in agreement with previous results, suggesting SO, which is rich in PUFAs, is elevating insulin resistance in mice (35), thus increasing risk for metabolic syndrome.

We also evaluated the effect of the oils used in this study on the expression of several genes. mRNA levels of the G6Pase gene were significantly lower for the C-PO group in comparison to other groups. However, there was no significant difference in the plasma glucose levels after an overnight fast among the groups. G6Pase is a liver and kidney membrane-bound enzyme that plays an important role in providing glucose during starvation. Starvation increases the level of the G6Pase mRNA expression by about 30% (26). It is possible that the C-PO enhances satiation and inhibits starvation pathways in comparison to other oils, explaining the lower G6Pase mRNA levels.

mRNA levels of the SREBP-1c gene was also found to be significantly lower for the C-PO group in comparison to other diets. SREBP-1c is a transcription factor that regulates lipid homeostasis. It controls expression of various enzymes required for endogenous cholesterol, fatty acid (FA), triacylglycerol, and phospholipid synthesis (36), including FAS and ACC enzymes (both these proteins are involved in fatty acids synthesis). Therefore, a correlation is expected between SREBP-1c mRNA levels and ACC and FAS expression with lower SREBP-1c levels leading to lower ACC and FAS expression. The ACC protein is active when not phosphorylated. It appears that lower active levels of ACC lead to a higher p-ACC/ACC protein ratio and lower fatty acid content in the liver. However, the p-ACC/ACC protein ratio was similar to the other groups, as well as liver lipid weight which was not different from the other groups. As expected, FAS mRNA levels were significantly lower for the C-PO group than for other groups. FAS plays a central role in de novo lipogenesis. Our results have shown that C-PO led to significantly reduce the m RNA FAS expression compared to the other diets including OO which is also high oleic oil. This may indicate that C-PO has potential to reduce the lipogenesis and protect against fatty liver development. We believe further examination is required.

No significant differences were observed in p-AMPK/AMPK protein ratio among the groups or in the activity levels of the ACC protein. However, a Student’s *t*-test revealed significant differences in AMPK levels between the C-PO and PO groups (*P* < 0.05). AMPK plays a role in cellular energy homeostasis as well as in SREBP-1c regulation. When cell energy is decreased, there is an increase in the active form of AMPK enzyme (p-AMPK) resulting in an increased p-AMPK/AMPK protein ratio. An increase in the p-AMPK/AMPK protein ratio decreases cellular anabolic processes and increases catabolic processes. Therefore, decreases in SREBP-1c gene expression and ACC activity were expected.

SIRT1 relative protein levels were similar among the groups. SITRT1 deacetylates proteins. AMPK enhances SIRT1 activity (via synthesis of NAD+), thus promoting protein deacetylation. SIRT1 also activates AMPK through LKB1 deacetylation. In fact, AMPK and SIRT1 create a negative feedback loop by affecting each other’s activation (37, 38). Therefore, a correlation in their expression is expected. However, no significant difference was found in SIRT1 levels between the C-PO and PO groups (Student’s *t*-test), as was observed for AMPK levels.

CD36 mRNA levels were higher in the OO group and lower in the C-PO group (*P* < 0.05). Generally, gene expression is a good indicator of protein expression in the body. Therefore, it was expected that the CD36 protein level would be impacted accordingly. Yet, there was no significant difference in the CD36 protein levels between the groups. We can only speculate about the lack of this correlation that both transcription and translation may be coordinately regulated, whereas there is additional regulation at the level of protein degradation. To the best of our experience and knowledge, there is not always a correlation between the level of expression mRNA and protein level of many genes including as we reported in the case of CD36 (39). It is possible that OO consumption inhibits mRNA translation, which could explain normal protein levels despite higher mRNA levels. Also, in our experiment, there was no correlation found between CD36 protein levels and liver lipid accumulation. CD36 protein levels were similar between the four diets. However, there were differences found in liver lipid accumulation among the groups. Studies have associated CD36 deficiency with defective fatty acid and glucose metabolism in hypertensive rodents (25, 40). In addition, studies have shown that CD36 overexpression enhances free fatty acid (FFA) uptake by hepatocytes (41, 42). All of our diets had standard fat percentage (as recommended by AIN-93 M diet). Hence, lipids accumulation in the liver can be attributed to other mechanisms, rather than CD36 expression. In this work, we show that the oils affected CD36 mRNA expression differently, with the C-PO group the expression being the lowest. We speculate that C-PO may lead to improved metabolic syndrome and carbohydrate metabolism. Further research is required.

C-PO and OO have similar lipid profiles (MUFA, PUFA, and saturated fat). They both are high in oleic acid which is considered to be beneficial in carbohydrate and lipid metabolism. Notably, our findings were different for both oils. This may be attributed to different linoleic/α-linolenic acid ratios or possibly related to other components in the oils. For example, studies show that extra virgin OO is rich in microcomponents that contribute to better lipid profiles and are not present in other oils rich in oleic acid (43).

In summary, our results suggest that in mice SO favors lipid accumulation in adipose tissue rather than in liver tissue, as well as causes decreased insulin sensitivity. Mice fed an OO diet had lower plasma TG and increased CD36 gene expression. The C-PO group presented lower mRNA levels for all examined genes: SREBP-1c, FAS, G6Pase, and CD36. There was no significant difference in weight gain, food intake, cholesterol and HDL cholesterol levels, ACC, SIRT1, AMPK, and CD36 protein levels and liver function among the four groups.

Overall, in our experiment, all the diets, including the same fat percentage, led to similar metabolic effects, with few nuanced differences. It appears that diets with the same fat percentage may lead to the similar health effects. Similar results were received in the study that examined high-fat diets composed of palm stearin and OO in mice. Both diets equally exacerbated liver inflammatory damage and metabolic stress (44). It seems that as long as fat is consumed in moderation, oil types may play a lesser role in the metabolism of healthy individuals. This finding has the potential to increase flexibility in choosing oil types for consumption.

It is important to note that in the SO group, a decrease in insulin sensitivity was observed in mice with only 4% (w/w) of SO intake. As a result, we believe that this oil might not be the best variety for individuals with prediabetes, diabetic mellitus. and people at the risk of developing type 2 diabetes. In addition, it seems that, in mice, 4% (w/w) of peanut oils (C-PO, PO) and OO promote lipid accumulation in liver, which maybe should be taken into consideration in people with fatty liver. However, it should be noted that fatty acid metabolism in mice differs from humans; therefore, some of the results seen in mice might not have the same effect on humans and further investigation is required. Finally, for C-PO, a new peanut variety, further investigation is suggested, as it can become an additional dietary source of oleic acid.
